# Physicochemical Properties of Sago- and Corn Flour-Based Rice Analogues Fortified with Black-Eyed Bean Flour and Skimmed Milk Powder

**DOI:** 10.17113/ftb.62.04.24.8357

**Published:** 2024-12

**Authors:** Noer Abyor Handayani, Siswo Sumardiono, Aprilina Purbasari, Alfan Fatir Fatikah, Imam Muda Alhakim

**Affiliations:** Department of Chemical Engineering, Faculty of Engineering, Universitas Diponegoro, Prof. Soedarto, SH Street, Tembalang, Semarang, 50275, Central Java, Indonesia

**Keywords:** rice analogue, fortification, corn flour, sago flour, hot extrusion

## Abstract

**Research background:**

With the increasing consumption of food commodities, particularly rice, and the substantial volume of food imports in Indonesia, there is an increasing need to explore alternative food sources. Rice analogues emerge as a potential substitute for traditional rice, serving as a viable staple food option. The aim of this study is to investigate the effect of the composition of raw material, namely sago and corn flour, on the physicochemical properties and consumer acceptance of rice analogues.

**Experimental approach:**

The rice analogues were produced using the hot extrusion method. The nutritional content (protein, carbohydrate, fat, moisture, fibre and ash) of the product was then analysed. Thermal stability and morphological properties were determined using differential scanning calorimetry (DSC) and scanning electron microscopy (SEM), respectively. A consumer acceptance test including taste, texture, aroma and colour was also carried out to evaluate the quality of cooked rice analogues.

**Results and conclusion:**

The results showed that the rice analogue produced by hot extrusion at 70 °C with black-eyed beans (15 % *m*/*m*) and skimmed milk powder (0.5 % *m*/*m*) had the most significant nutritional improvements such as increased content of protein, fat, carbohydrate, moisture content, ash and fibre. However, it should be noted that increasing the extrusion temperature above 70 °C meant that both density and hardness could no longer be controlled due to gelatinisation. Furthermore, 30 participants in the consumer acceptance test rated the texture, aroma, taste and colour positively, underlining the potential of rice analogue as a nutritious and attractive alternative to natural rice. The rice analogue made from modified sago with additional ingredients of corn and black-eyed beans has similar properties to natural rice.

**Novelty and scientific contribution:**

The combination of sago, corn and black-eyed bean flour as the main ingredients of the rice analogue is a novelty of this study. Furthermore, its nutritional profile exceeds that of natural rice, making it a viable and acceptable alternative in times of rice scarcity.

## INTRODUCTION

Food plays an important role in sustaining human life as it serves as a primary source of energy for humans. In Indonesia, rice is the predominant staple food and dependence on this dietary source is high (*1*). Data from the Indonesian Central Statistics Agency (BPS) show that the country imported 3.06 million tonnes of rice in 2023, the highest record in the last five years (*1*). Despite this dependence on rice, the culture of rice consumption in Indonesia is deeply rooted and resistant to change, even with a growing population. Notably, Indonesia leads the world with an average per capita consumption of approx. 130 kg per year, followed by Thailand (90 kg), Malaysia (80 kg) and Japan (45 kg) (*2*). Rice consumption in Indonesia is forecast to gradually increase by 1.5 % per capita in 2025 and 2 % by 2045 (*2*). If such a scenario is repeated in the future, it could pose significant challenges to food security and impact various aspects of human life.

Food diversification, particularly addressing rice shortages, has attracted considerable attention to increase the resilience of food security (*3*). Rice analogue, a notable food diversification initiative, serves as a carbohydrate source derived from non-paddy plants such as tubers, cereals and other crops that are still underutilised despite their abundance (*4*). The goal of rice analogue is to mimic or even surpass the properties of natural rice in terms of carbohydrate content while maintaining a low glycaemic index. To make non-rice foods attractive to the general public, the production of rice analogues with improved physical properties requires a suitable process (*5*).

The quality of rice analogues is closely related to their composition, which affects their physical properties, texture and nutritional value (*6*). Rice analogues can be produced from various native raw materials, including sago, sorghum, tubers and corn. Sago (*Metroxylon sagu* Rottb) is a promising carbohydrate source for replacing rice due to its high carbohydrate content of approx. 78 % (*2*, *7*). Indonesia has a significant area of sago cultivation estimated at 1.2 million acres, with an annual yield ranging from 8.4 to 13.6 million tonnes. Sago productivity can reach an impressive 25 tonnes per hectare per year, surpassing other starch crops. However, the high amylopectin content of sago can lead to undesirable stickiness and a hard texture of the final rice analogue produced (*1*).

To improve its properties, sago starch can be modified by adding various other raw materials. Another natural raw material that can serve as an additional component in rice analogues is corn starch (*Zea mays* L.). Corn, a starchy vegetable and cereal grain, has been a dietary staple food worldwide for generations. It is not only rich in fibre but also in vitamins and minerals. Its fibre content contributes to the well-distributed starch mixture in the rice analogue and improves its structure (*7*). By breaking the covalent and non-covalent bonds between carbohydrates and fibre in the processed rice analogue, corn also facilitates the formation of smaller and more soluble molecular fragments that help improve the digestion of the rice analogue. Corn is also high in starch, essential minerals and β-carotene (provitamin A), all of which are beneficial to human health (*8*).

The nutritional content of rice analogues requires evaluation and improvement. Sumardiono *et al*. (*9*) conducted a study on the development of rice analogues using sago flour, corn flour and mung bean flour. Since Indonesia only produces 284 260 tonnes of mung beans per year, black-eyed beans (*Vigna unguiculata* L. Walp) are added to the rice analogue as a vegetable protein source to increase productivity to 494 506 tonnes per year (*10*).

Black-eyed beans contain protein, carbohydrates and fibre, approx. 22–33, 33–59.6 and 2–3 %, respectively. This type of legume also contains a number of antioxidants, minerals (including phosphorus and iron) and vitamins (such as A, B1 and C) that greatly enhance the nutritional profile of rice analogues (*11*). Moreover, the production of rice analogues must focus on the improvement of flavour and overall quality. The addition of skimmed milk powder aims to bind the rice analogue and give it a texture that imparts a savoury taste. Skimmed milk powder also contains calcium, which promotes healthy bones and helps prevent osteoporosis (*12*).

In the production of rice analogues, the extrusion method is used to produce a solid food product from powdered materials and emulsifiers (water and oil). This process contributes to high-quality results and is widely used in the food industry (*13*). During extrusion, reactions occur between starch and other raw ingredients inside the extruder tube and mould (*12*).

Numerous studies have been conducted by various researchers (*2*, *4*-*7*, *14*), focusing on the development of the physical and qualitative aspects of rice analogues; however, research on consumer acceptance remains limited. The production of rice analogues that are similar in properties and taste to natural rice also remains an ongoing challenge. The aim of this study is to investigate the effects of using sago and corn flour in the hot extrusion process on the nutritional value, the effects of fortification with black-eyed beans as a treatment combination, the physical properties of the product using scanning electron microscope (SEM) and the consumer acceptability of rice analogue. Additionally, the results of differential scanning calorimetry (DSC) are also discussed in this study.

## MATERIALS AND METHODS

### Materials

The materials for the study were obtained as follows: sago flour was procured from CV. Sagu Tani (Bogor, Indonesia), while corn flour was obtained from PT Ega Multi Cipta/Egafood (Banten, Indonesia). Black-eyed beans were purchased from Pasar Ki Lemah Duwur Bangkalan (Madura, Indonesia) and manually processed into powder. Skimmed milk powder was supplied by NZMP (Auckland, New Zealand). Glycerol monostearate was purchased from CV. Multi Jaya Kimia (Tangerang, Indonesia). Distilled water and cooking oil were purchased locally from CV. Indrasari (Semarang, Indonesia).

### Rice analogue production

The rice analogue was produced using the hot extrusion process according to Sumardiono *et al.* (*9*). First, *m*(sago):*m*(corn flour)=1:1 were mixed. Distilled water, accounting for 50 % of the total mixed flour mass, was then added to this mixture. Glycerol monostearate (2 % *m*/*m*) was also added to reduce the water solubility index and minimise friction heat during extrusion (*5*). Black-eyed bean flour and skimmed milk powder were added into the mixture in different mass fractions, as shown in Table S1. To facilitate the mixing process, the entire composition was mixed with a food mixer (HR7922/90; Phillips, Surabaya, Indonesia) for 20 min, adding 10 drops of cooking oil. Before extrusion, the mixed dough was steamed at a temperature from 80 to 90 °C for 30 min to precondition it. The steamed dough was then fed to the extruder (hot extrusion) at the temperatures indicated in Table S1. The extrusion process was carried out using the MPF-24 Small Batch Twin Screw Extruder from Baker Perkins (Warren, MI, USA). In this study, the extrusion was carried out at three different temperatures (50, 70 and 90 °C), while maintaining the same set of sample formulations throughout. In addition, their influence on the nutritional content of rice analogue was to be observed in this experiment using a proximate test.

### Proximate analysis

The chemical analysis was also carried out to determine multiple components, including protein, fat, carbohydrates, moisture, ash and fibre (*15*). Each parameter was determined using these methods: the protein content was determined using the Kjedahl method, which includes three stages: (*i*) digestion (where the sample is decomposed using sulfuric acid to convert nitrogen to ammonium sulfate), (*ii*) distillation (where ammonium is distilled into boric acid solutions), and (*iii*) titration to determine the amount of nitrogen using standard acid solution (*16*). The protein content was determined from the nitrogen amount of the rice analogue (conversion factor of 6.25). The fat content was measured using the extraction method (SNI 289101) (*15*). The sample was placed in a Soxhlet apparatus and hexane was used as a solvent. The fat was extracted in several cycles and once the solvent had evaporated, the remaining fat in the apparatus was weighed and calculated on the basis of the initial sample mass. The moisture content was determined in an oven SH-DO-90FH (SH Scientific, Jakarta, Indonesia) at 105 °C for 3 h by measuring the initial and final mass of the sample (*9*). The ash content was determined using an electric furnace (maximum 550 °C; muffle furnace Nabertherm, Lilienthal, Germany) until the ashing process was complete. The difference in sample mass before and after burning was then calculated (*15*). The carbohydrates were determined by hydrolysis, in which carbohydrates are converted into monosaccharides. These monosaccharides reduced Cu(II) to Cu(I). The excess Cu(II) was analysed using the iodometry method. The carbohydrate content was then calculated as a difference between the initial and the reduced amount of Cu(II) (*15*). The fibre content was determined using AOAC method 978.10, which uses sequential acid-alkaline extraction (*17*). With this method, protein, sugar, starch, lipids, carbohydrates and linin were removed and crude fibre was identified as residue. The residue remaining after extraction, consisting of crude fibre, was weighed and the fibre content was expressed as a percentage of the initial sample mass (*17*).

### Consumer acceptance analysis

The consumer acceptance analysis was conducted to evaluate the quality of cooked rice analogue, including taste, texture, aroma and colour. The score range of 1 to 4 was used for this evaluation, where panellists could give a score of 1 (unpleasant), 2 (quite pleasant), 3 (pleasant) and 4 (very pleasant). Thirty people participated in this study (age range: 21–25, 12 males and 18 females) at Chemical Engineering Department, Engineering Faculty, Diponegoro University, Semarang, Indonesia. In this study, all panellists agreed to participate by signing an informed consent form. Each determination analysis was carried out in triplicate and a hedonic test (based on four parameters) was performed according to Destarianto *et al.* (*18*) to distinguish the preference levels of 30 panellists for each product.

### Surface electron microscopy

The physical analysis focused on determining surface micrograph characteristics using a scanning electron microscope (SEM, XL series 30; Philips, Rotterdam, the Netherlands) (*10*). The SEM analysis was conducted at a magnification of 500× on rice analogue with *w*(black-eyed bean)=15 % and *w*(skimmed milk)=0.5 % and extrusion temperatures of 50, 70 and 90 °C (sample formulations 2, 11 and 20).

### Differential scanning calorimetry

Thermal analysis was carried out by determining the glass transition temperature using differential scanning calorimetry (DSC, NEXTA STA200RV with Real View sample observation; Hitachi, Seoul, South Korea) (*8*). This analysis is necessary to determine the thermal stability of the rice analogue, as rice is usually cooked at relatively high temperatures before consumption.

### Statistical analysis

The Duncan’s multiple-range test was used to evaluate the main differences when the mean values of the main effects or interactions were statistically significant (p≤0.05). Mean values and standard deviations were calculated in each case using Microsoft Excel (Microsoft Office 2019, Redmond, WA, USA) and IBM SPSS v. 25 package programme (*19*). The findings were then compared with those of an earlier study by Sumardiono *et al*. (*6*).

## RESULTS AND DISCUSSION

### Nutritional properties of rice analogue

In this study a proximate analysis was performed to determine the nutritional content of rice analogues. The results of the proximate analysis of protein, fat, carbohydrate, moisture, ash and fibre mass fraction of rice analogues are shown in Table 1.

**Table 1:** Results of proximate analysis of rice analogue with different mass fractions of black-eyed bean and skimmed milk

Sample	*w*/%						
formulation	Protein	Fat	Carbohydrate	Moisture	Ash	Fibre	*t*/°C
1	(6.4±0.2)^c^	(2.28±0.01)^a^	(82.8±0.3)^b^	(7.73±0.03)^b^	(0.860±0.005)^a^	(7.2±0.3)^a^	50
2	(6.62±0.03)^c^	(2.75±0.02)^b^	(81.3±0.4)^a^	(8.44±0.04)^c^	(0.980±0.004)^b^	(7.5±0.9)^a^	
3	(6.85±0.03)^d^	(2.34±0.02)^a^	(82.1±0.4)^b^	(7.87±0.04)^c^	(0.880±0.004)^a^	(9.3±0.4)^c^	
4	(6.43±0.03)^c^	(2.05±0.02)^a^	(82.5±0.4)^b^	(8.18±0.03)^c^	(0.850±0.006)^a^	(8.4±0.9)^b^	
5	(6.80±0.03)^d^	(2.17±0.03)^a^	(82.2±0.6)^b^	(7.83±0.04)^c^	(0.970±0.006)^b^	(7.6±0.6)^a^	
6	(7.55±0.02)^d^	(2.11±0.03)^a^	(82.3±0.6)^b^	(7.36±0.04)^b^	(0.680±0.007)^a^	(7.2±0.7)^a^	
7	(6.31±0.02)^c^	(2.07±0.03)^a^	(81.9±0.6)^a^	(9.01±0.04)^c^	(0.720±0.007)^a^	(8.4±0.6)^b^	
8	(6.850±0.005)^d^	(2.35±0.03)^a^	(81.7±0.6)^a^	(8.22±0.04)^c^	(0.880±0.008)^b^	(8.2±0.6)^b^	
9	(6.730±0.004)^c^	(2.16±0.03)^a^	(83.0±0.7)^c^	(7.32±0.04)^b^	(0.750±0.008)^a^	(7.4±0.7)^a^	
10	(6.59±0.01)^c^	(2.18±0.03)^a^	(82.0±0.7)^b^	(7.54±0.04)^b^	(0.760±0.008)^a^	(7.4±0.7)^a^	70
11	(5.720±0.004)^b^	(2.46±0.04)^a^	(82.9±0.8)^b^	(7.12±0.04)^a^	(0.980±0.009)^b^	(8.2±0.6)^b^	
12	(6.01±0.01)^b^	(2.41±0.04)^a^	(83.3±0.8)^c^	(7.12±0.04)^a^	(1.150±0.009)^b^	(9.1±0.8)^c^	
13	(5.720±0.003)^b^	(3.12±0.04)^b^	(83.5±0.4)^c^	(7.12±0.04)^a^	(0.75±0.01)^a^	(9.3±0.9)^c^	
14	(6.290±0.003)^c^	(2.52±0.02)^b^	(81.7±0.2)^a^	(7.56±0.04)^b^	(1.18±0.01)^c^	(8.4±0.2)^b^	
15	(6.87±0.01)^d^	(2.65±0.02)^b^	(82.4±0.2)^b^	(7.12±0.04)^a^	(0.89±0.01)^b^	(8.2±0.2)^b^	
16	(5.560±0.003)^a^	(2.45±0.06)^a^	(82.5±0.2)^b^	(7.44±0.04)^b^	(0.85±0.01)^a^	(8.7±0.4)^b^	
17	(5.36±0.02)^a^	(2.45±0.06)^a^	(83.7±0.2)^c^	(7.25±0.05)^b^	(1.13±0.01)^b^	(7.9±0.2)^a^	
18	(6.220±0.003)^c^	(2.76±0.06)^b^	(83.8±0.3)^c^	(7.02±0.05)^a^	(1.07±0.02)^b^	(9.3±1.0)^c^	
19	(5.15±0.03)^a^	(2.55±0.01)^b^	(82.9±0.3)^b^	(6.91±0.05)^a^	(0.93±0.02)^b^	(7.4±0.3)^a^	90
20	(5.06±0.03)^a^	(2.25±0.01)^a^	(84.4±0.3)^d^	(6.85±0.05)^a^	(1.42±0.02)^c^	(9.3±0.3)^c^	
21	(5.83±0.03)^b^	(2.3±0.01)^a^	(83.1±0.3)^c^	(6.23±0.05)^a^	(1.33±0.02)^c^	(9.1±1.0)^c^	
22	(5.69±0.02)^b^	(3.13±0.01)^b^	(84.3±0.3)^d^	(8.12±0.06)^c^	(1.52±0.02)^c^	(7.4±0.3)^a^	
23	(5.87±0.01)^b^	(2.54±0.04)^b^	(81.5±0.3)^a^	(7.04±0.06)^a^	(0.97±0.02)^b^	(7.6±0.3)^a^	
24	(5.46±0.02)^a^	(2.3±0.04)^a^	(83.6±0.4)^c^	(7.13±0.05)^a^	(1.13±0.03)^b^	(8.9±0.9)^b^	
25	(5.17±0.02)^a^	(2.45±0.05)^a^	(84.2±0.4)^d^	(7.03±0.05)^a^	(1.18±0.04)^c^	(8.4±0.9)^b^	
26	(5.07±0.01)^a^	(2.54±0.05)^b^	(84.2±0.4)^d^	(6.95±0.04)^a^	(1.22±0.05)^c^	(8.5±0.9)^b^	
27	(5.16±0.02)^a^	(2.02±0.05)^a^	(84.6±0.4)^d^	(7.36±0.05)^b^	(1.25±0.05)^c^	(8.6±0.4)^b^	
IR64*	(7.18±0.04)	(0.360±0.004)	(78.1±0.1)	N/A	(0.560±0.004)	(3.29±0.04)	

Values are expressed as mean±standard deviation, *N*=3. Values followed by a different letter in the same column are statistically significantly different (p<0.05). *Data were collected from previous study by Sumardiono *et al*. (*9*). The composition of samples 1 to 27 is given in Table S1

### Protein content

The analysis showed that the highest protein mass fraction (7.55 %) was in sample formulation 6 and the lowest in sample formulation 20 (5.06 %) (Table 1). In a previous study, the protein mass fraction of rice analogues containing mung beans was reported to be between 2.73 and 4.83 % (*9*). The protein mass fraction in the new formulation of rice analogue with black-eyed bean flour was higher than that of the previous formulation. The addition of black-eyed beans directly affects the protein content of the rice analogue as they are known for their high protein content. In comparison, natural rice (IR64) usually contains about 7.18 % protein (*9*). However, the protein mass fraction of rice analogue is lower than that of natural rice due to the specific composition of black-eyed beans. To achieve the recommended protein intake, other protein sources such as eggs, meat, fish and vegetables can be included in the diet. Protein is a macromolecule made up of carbon, hydrogen, oxygen and nitrogen atoms and plays an important role in cell repair, transport and immune protection in the human body. According to Harvard Medical School, the recommended intake of protein is modest, 0.8 g/kg body mass or at least 53 g/day for adults aged 17 to 80 years (*5*). Rice analogues with *w*(black-eyed bean)=20 % and *w*(skimmed milk)=1 % (sample formulation 6) can help meet 40 % of this daily requirement if consumed three times a day. It is important to note that excessive protein consumption can lead to adverse effects such as obesity. Excess protein is stored in the body as triglycerides, which can contribute to obesity (*20*).

### Fat content

The highest fat mass fraction (3.13 %) was observed in sample formulation 22, while the lowest was recorded in sample formulation 27 at 2.02 % (Table 1). In another paper, the fat mass fraction of rice analogues from mung beans was reported to be between 1.35 and 2.28 % (*9*). The new rice analogue with black-eyed bean flour had a higher fat mass fraction than the previous formulation. This variation in fat mass fraction could be attributed to the different compositions of the rice analogues. It is worth noting that the fat mass fraction of rice analogues is higher than that of IR64 rice, which contains approx. 0.36 % fat. This difference is primarily due to the addition of black-eyed bean flour and skimmed milk powder in the raw materials. Among legumes, black-eyed beans have a relatively high fat content, with a significant amount of this fat consisting of unsaturated fatty acids (*21*). Additionally, the fat in black-eyed beans can help reduce the risk of hypocholesterolaemia by decreasing LDL cholesterol while increasing HDL cholesterol levels, thus reducing the likelihood of atherosclerosis and cardiovascular disease (*22*). Foods with a higher fat content tend to slow down gastric emptying and digestion in the small intestine, resulting in meals with a lower glycaemic index (*23*).

### Carbohydrate content

Among the experimental formulations, the highest carbohydrate content (84.56 %) was observed in sample formulation 27, while the lowest (81.31 %) was found in sample formulation 2 (Table 1). In a previous work, it was described that the carbohydrate mass fraction of rice analogue with mung beans ranged from 80.73 to 84.31 % (*9*). The carbohydrate content in the new composition of rice analogue with black-eyed bean flour was higher than that of commercial rice (IR64), see Table 1. The carbohydrate content in the rice analogue is influenced by a combination of sago flour, peanut flour, skimmed milk powder flour and corn starch, with sago flour and corn starch being the dominant components. Sago flour and corn starch are known for their high carbohydrate mass fraction: 84.7 and 73 %, respectively (*23*, *24*). The extensive use of sago flour and corn starch in the mixed raw components contributes to the high carbohydrate amounts in the rice analogue. For comparison, the standard carbohydrate mass fraction of IR64 rice is approx. 77.1 % (*6*). According to the standards of the Ministry of Health, the carbohydrate content of rice analogues is suitable for daily consumption. Amylopectin and amylose are examples of these carbohydrate types. The amount of amylose in a carbohydrate has a direct impact on the glycaemic index rating of the food. Sago flour, used as a carbohydrate source in the production of rice analogues, offers several advantages over other commodities. It has a low glycaemic index of 43.25, has a high amylose content and contains beneficial antioxidants (*25*).

### Moisture mass fraction

The experiments showed that the highest moisture mass fraction (9.01 %) was found in sample formulation 7 and the lowest (6.23 %) in sample formulation 21 (Table 1). In another study (*9*), the moisture mass fraction of the rice analogue with mung beans ranged from 10.17 to 10.55 %. The moisture mass fraction in the new formulation of rice analogue with black-eyed bean flour was lower than in a previously published formulation with mug beans. It is worth noting that the moisture mass fractions of the rice analogue in this study were below the standard for safe rice storage, which is usually less than 14 %. It is important to keep the water content in conventional rice (IR64) below 14 % (*m*/*m*) to prevent the growth of moulds that can thrive on the grains. If rice exceeds this moisture threshold, typically above 14 % (*m*/*m*), it can become more brittle, making it susceptible to damage and spoilage during storage (*5*).

### Ash mass fraction

The analysis of the ash content in rice analogues from sago, corn, black-eyed bean and skimmed milk powder yielded values between 0.68 and 1.52 % (Table 1). The ash mass fraction of the rice analogue was higher than that of conventional rice (IR64), which is about 0.56 %. In a previous study, the ash mass fraction of rice analogue with mung beans was reported to be between 1.31 and 1.65 % (*9*). The ash mass fraction of the new formulation of rice analogue with black-eyed bean flour was lower than of the previous formulation. The ash content of rice analogue serves as an indicator of the amount of minerals. Ash in this context refers to the mineral residues that remain after combustion at high temperature. Higher mass fractions of ash residue indicate that the rice analogue has been successfully enriched with minerals, making it a potentially healthier option due to the presence of these minerals necessary for human health (*5*). However, this finding was lower than in a previous study, which indicated that black-eyed bean flour has a lower mineral content than mung bean flour.

### Fibre mass fraction

The highest fibre mass fraction (9.3 %) was found in sample formulations 3, 13, 18 and 20 and the lowest (7.2 %) in sample formulation 1 and 6 (Table 1). Another paper described that the fibre mass fraction of rice analogues with mung beans ranged from 2.06 to 5.57 % (*9*). The fibre content of the new formulation of rice analogues with black-eyed bean flour was lower than that of the previous formulation. The main sources of fibre in rice analogues are corn flour and black-eyed bean flour. In conventional rice, the fibre mass fraction is typically between 0.4 and 0.6 % (*26*). In contrast, rice analogues contain a significantly higher fibre content than conventional rice. According to Kanetro *et al.* (*27*), rice analogue with black-eyed beans has a lower glycaemic index than conventional rice. This is attributed to the significantly higher fibre content due to the black-eyed beans. Dietary fibre plays a crucial role in slowing down the absorption sugar and cholesterol during digestion (*28*). Furthermore, the protein in black-eyed beans can also influence the glycaemic index values. Legume proteins, such as those found in black-eyed beans, contain high amounts of the amino acid arginine, which is known to promote insulin release (*27*). However, in previous studies by Sumardiono *et al*. (*6*), mung beans were found to contain 16 g/100 g of dietary fibre, while black-eyed beans contain only 7 g/100 g.

### Effect of extrusion temperature on nutritional properties

Table 1 shows the effect of extrusion temperature on the nutritional properties of rice analogues. The protein content in the rice analogue samples was 6.62 (sample 2), 5.720 (sample 11) and 5.06 % (sample 20) at extrusion temperatures of 50, 70 and 90 °C, respectively. Notably, the lowest protein mass fraction was observed at 90 °C. Interestingly, the experiments showed that increasing the extrusion temperature led to a decrease in the protein mass fraction in the rice analogue. This decrease in protein content can be attributed to the high temperature during extrusion, which can damage the protein structure (*29*). Additionally, studies by Alyani *et al.* (*30*) suggest that protein content can decrease due to denaturation of protein coagulation. Higher temperatures lead to more extensive denaturation of proteins, with denaturation occurring already at 50 °C (*30*). Denaturation leads to changes or damage to the secondary, tertiary and quaternary structures of proteins (*31*). From this, it can be concluded that higher extrusion temperatures lead to lower protein content in the final product.

According to Table 1, the carbohydrate mass fraction in the rice analogue samples ranged from 81.3 to 84.4 %. The highest carbohydrate mass fraction was obtained at an extrusion temperature of 90 °C, while the lowest was obtained at 50 °C. The experiments showed a direct proportional relationship between carbohydrate mass fraction and extrusion temperature, with higher temperatures leading to higher carbohydrate mass fraction. This phenomenon can be attributed to the separation of amylose and amylopectin chains in the rice analogues, which affects the amount of these molecules. Higher temperatures facilitate the separation of amylose and amylopectin molecules, resulting in a higher content of both amylose and amylopectin (*32*). Additionally, the branching and crosslinking of molecules are more pronounced in rice analogues than in natural rice because the phosphate groups penetrate the granules and form covalent bonds with the starch molecules, resulting in larger molecules (*33*).

The fat mass fraction of the rice analogue was between 2.05 and 3.13 %, as shown in Table 1. The highest fat mass fraction was obtained at an extrusion temperature of 90 °C and the lowest at 50 °C. According to Shelton and Lee (*33*), fat consists of mixed triglycerides, which are esters of glycerol and long-chain fatty acids. The hydrolysis of fat leads to the formation of three molecules of long-chain fatty acids and one molecule of glycerol. Increasing the extrusion temperature during the process can result in a decreased fat content in the rice analogues. Temperature and processing time are factors in food processing that can cause a reduction in fat content in the final product, as fats are sensitive to heat and can degrade during processing (*34*).

The crude fibre mass fraction of the rice analogue was between 7.2 and 9.3 %, as shown in Table 1. The highest crude fibre mass fraction was observed at an extrusion temperature of 90 °C, while the lowest was at 50 °C. In the experiments, it was found that increasing the extrusion temperature the higher crude fibre content in the rice analogue was obtained. According to Palupi (*34*), higher extrusion temperatures lead to a lower moisture content and an increase in powdered carbohydrates (*35*). Fibre is a complex carbohydrate found in food, and the increase in the carbohydrate content in food is directly proportional to the crude fibre content. This relationship is also confirmed by previous studies showing that higher extrusion temperatures lead to higher carbohydrate content (*36*).

As shown in Table 1, the moisture mass fraction of the rice analogue ranged from 6.23 to 9.01 %. The highest moisture mass fraction was obtained at an extrusion temperature of 50 °C, while the lowest value was at 90 °C. One of the factors contributing to the decrease in moisture content in the food was the extrusion temperature. Higher temperatures cause higher water evaporation from the material (*37*). Additionally, the moisture mass fraction in the rice analogue decreases during extrusion due to the increased temperature and milling of the rice, which leads to structural changes and protein shrinkage, creating pressure that forces the water to escape, resulting in a decrease in moisture content (*38*). The proximate analysis of the influence of extrusion temperature on the rice analogues showed that protein, fat and moisture mass fraction decreased with increasing temperature, while carbohydrate and crude fibre mass fraction increased. The optimum temperature for the experiment was therefore 70 °C.

### Morphological properties of rice analogue

**Fig. 1:**
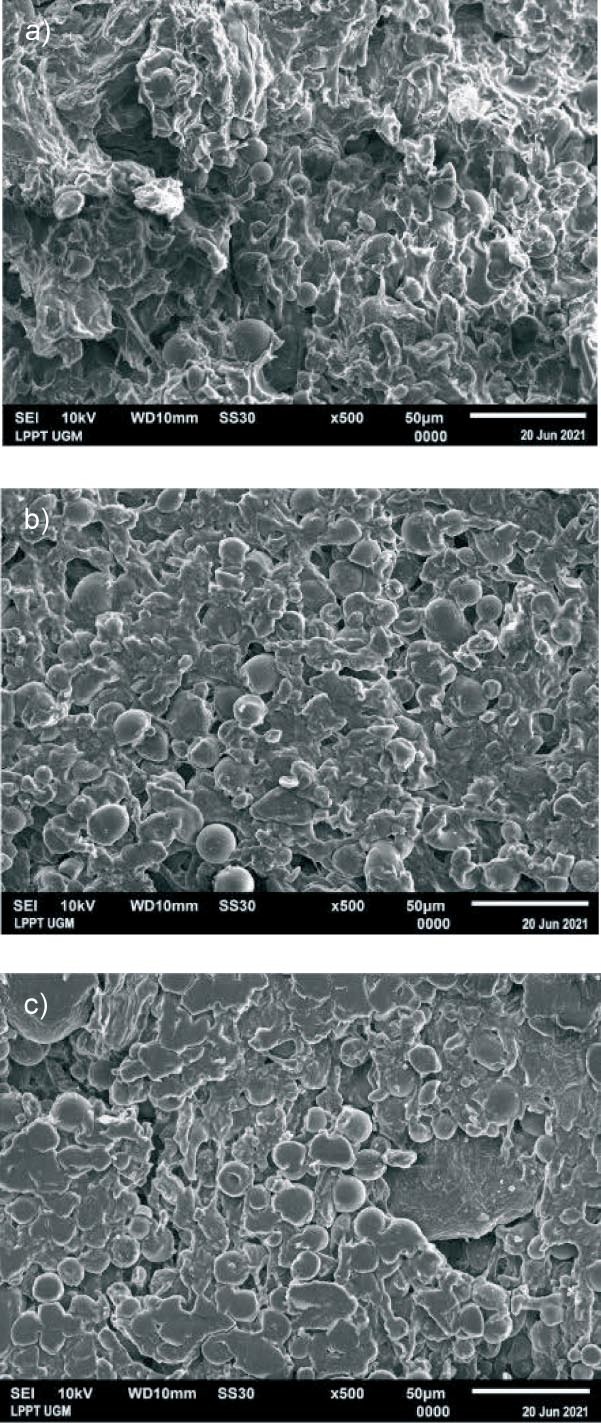
Results of scanning electron microcopy of sample formulation containing *w*(black-eyed bean)=15 % and *w*(skimmed milk)=0.5 % extruded at temperature: a) 50 (sample 2), b) 70 (sample 11) and c) 90 °C (sample 20)

In sample formulation 2 (Fig. 1a), which was extruded at 50 °C, the starch granules appeared swollen due to gelatinisation, but remained mainly intact. Some starch granules showed signs of damage due to gelatinisation and subsequent retrogradation. These damaged starch granules were not tightly bound to others, creating voids in the structure. This could compromise the physical integrity of the rice analogue, making it fragile and prone to breakage (*7*). During gelatinisation, water penetrates the starch granules, leading to the breakdown of intramolecular hydrogen bonds. These hydrogen bonds play a crucial role in maintaining the integrity of the granules. The presence of free hydroxyl groups in the starch absorbs water and facilitates the formation of starch granules (*39*).

In the SEM image of the sample formulation 11 (Fig. 1b), where the rice analogue was extruded at 70 °C, the number of intact granules appears to be reduced due to damage. While some granules still appeared intact, there were signs of partial damage and they appeared to be more tightly bound together. This bonded, swollen granule structure likely contributed to the increased strength of the rice analogue (*7*). SEM analysis in Fig. 1b also showed that the granule particles in sample formulation 11 were denser than in sample formulation 2, which is consistent with the higher fibre content in formulation 11. According to Richana and Sunarti (*39*), the fibre content influences the water absorption by starch granules. In cases with a lower fibre content, starch granules absorb less water, which leads to swelling without complete breakdown, making the starch granules look more intact.

In the SEM image of sample formulation 20 (Fig. 1c), where the rice analogue was extruded at 90 °C, the starch granules appear more fused. This suggests that retrogradation has occurred within the starch granules, resulting in significant fusion and cohesion of the starch, contributing to the increased strength and hardness of the rice analogue (*8*). This suggests that higher extrusion temperatures may affect the hardness of the rice analogue, with higher temperatures potentially leading to stronger particle bonding and greater hardness (*40*).

### Consumer acceptance evaluation

The organoleptic evaluation of the rice analogues involved a consumer acceptability test to evaluate parameters such as aroma, colour, texture and taste. In the food industry, flavour is a crucial parameter. Foods with overly intense or unpleasant flavour are often less desirable for consumption, especially staple foods like rice, particularly by Indonesian consumers. The flavour of rice analogues is influenced by various factors, including the extrusion process, residence time, extrusion temperature, moisture content, pressure and volatile compounds (*41*). Texture is another important property evaluated in organoleptic testing. It refers to the sensory properties of food that can be observed by visual inspection, touch and mouthfeel during consumption (*42*). Additionally, colour and taste are essential indicators that are often considered in organoleptic analysis. The taste of a food must match with the preferences of the target market or consumers. An unpleasant or unappealing taste can significantly reduce consumer interest in a product. Organoleptic tests are conducted to ensure that the rice analogue meets consumer standards and is accepted by potential consumers. These tests involved 30 subjects, who served as representatives of the consumer base, and were evaluated using the predefined parameters listed in Table S2.

### Aroma

**Fig. 2:**
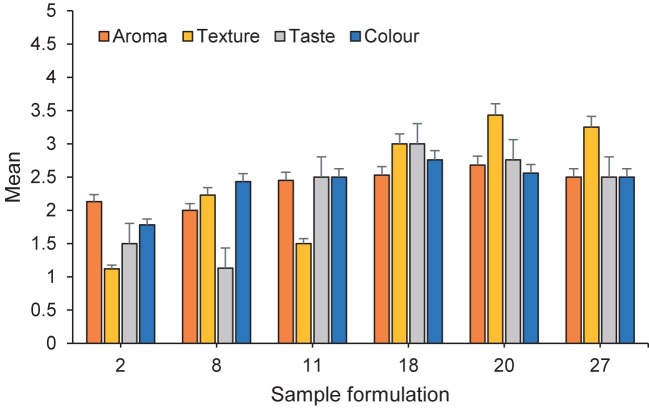
Organoleptic scores for aroma, texture, taste and colour of rice analogues

The average aroma scores for the different sample formulations varied, as shown in Fig. 2, The highest average scores were observed in sample formulations 20 and 27 with 2.68 and 2.50, respectively. Conversely, the lowest average was reported for sample formulations 2 and 8 at 2.13 and 2.00, respectively. Panellists observed that the aroma of the rice analogue was somewhat unpleasant, probably due to both the raw materials used and the processing methods. Unpleasant taste, such as sour taste, can be attributed to microbial activity, browning reactions and starch hydrolysis caused by the enzyme amylase. It is also important to control the addition of cooking oil, as improper storage and handling can lead to the breakdown of triglyceride bonds into glycerol and free fatty acids, resulting in a rancid lavour (*43*).

### Texture

Texture is a crucial aspect of food that can be perceived through visual, tactile and oral sensations (*42*). In this study, texture was tested involving 30 subjects and the results are shown in Fig. 2. Among the sample formulations tested, formulations 2 and 11 received the lowest average scores, while formulations 20 and 27 received the highest scores. The panellists described the texture of the cooked rice analogue as similar to conventional rice, which is sticky and soft after cooling. Such a texture is a result of the high proportion of sago flour in the raw materials used to produce the rice analogue. According to Putri *et al.* (*44*), a high sago flour content increases the amylopectin content in rice. Amylopectin, a type of carbohydrate, not only affects nutritional value but also determines the physical structure of cooked rice resulting from the cooking process (*43*). When the amylopectin content exceeds that of amylose, the cooked rice tends to become soft, like Japanese rice. A high amylopectin content in rice leads to stickiness, a glossy appearance, minimal expansion and lumpiness when cooled.

### Taste

In Fig. 2, the taste assessment by the panellists shows that sample formulations 18 and 27 received the highest scores among all formulations tested, while sample formulations 2 and 8 received average scores. The rice analogues were found to have a slightly different taste compared to conventional rice. The taste of rice analogues was described as slightly bitter and sour, which can be attributed to the relatively high proportion of sago flour in the raw materials used to make rice analogues. Sago generally has a neutral taste, but it can be affected if the storage and quality control of sago starch are not optimal. Improper storage conditions, particularly in damp environments, can lead to the growth of yeast and the development of a sour and musty smell, which in turn can affect the taste and aroma of the food (*43*).

### Colour

Fig. 2 shows that sample formulations 18 and 20 received the highest average scores, while sample formulations 2 and 8 received the lowest scores. Most panellsits positively evaluated the colour of the rice analogues. Rice analogue has a different colour than regular rice; it typically has a slightly brownish-white colour with cloudiness. This colouring of the rice analogue is primarily due to the sago flour, which undergoes enzymatic browning. Enzymatic browning is an oxidation reaction involving polyphenolase enzymes in the presence of oxygen. Polyphenols are oxidised to di-polycarbonyl or polycarbonyl compounds, resulting in a reddish-brown or reddish colour. Additionally, colour changes in rice can be attributed to the Maillard reaction, which occurs at high temperatures, such as during frying, roasting and cooking. In the production of rice analogues, treatments with high temperature are applied during steaming and extrusion, which contributes to the colour changes (*45*).

### Thermal stability analysis using differential scanning calorimetry

**Fig. 3:**
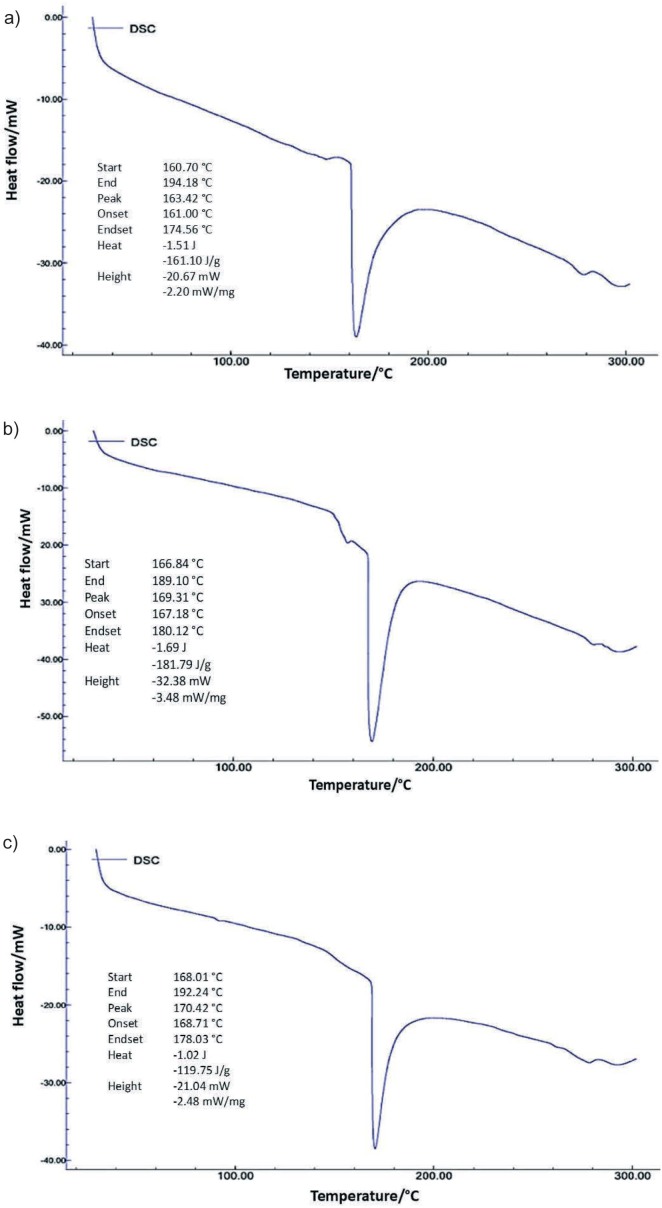
Thermal analysis of sample formulation containing *w*(black-eyed bean)=15 % and *w*(skimmed milk)=0.5 % extruded at temperature: a) 50 (sample 2), b) 70 (sample 11) and c) 90 °C (sample 20)

Fig. 3 shows the DSC test results for rice analogue samples of three different sample formulations: formulation 2 with an extrusion temperature of 50 °C (Fig. 3a), formulation 11 with an extrusion temperature of 70 °C (Fig. 3b) and formulation 20 with an extrusion temperature of 90 °C (Fig. 3c). The DSC results showed that formulations 2, 11 and 20 had peak temperatures of 163, 169 and 170 °C, respectively. In the starch gelatinisation, a peak temperature value represents the temperature at which starch crystals begin to melt (*46*). According to the data, formulation 20, which was processed at an extrusion temperature of 90 °C, had a higher gelatinisation temperature than formulations 2 and 11, which were extruded at 50 and 70 °C, respectively. It is also worth noting that higher extrusion temperatures are associated with greater starch gelatinisation (*8*). During extrusion, the starch granules swell and then retrograde. Retrograded starch tends to fuse and form stronger bonds, which could explain why the rice analogue, processed at 90 °C (formulation 20) had a higher gelatinisation temperature than the other formulations (*8*).

In formulations 2, 11 and 20, the addition of black-eyed beans led to higher protein mass fraction in the rice analogues. The increased protein mass fraction led to a delayed gelatinisation, which is reflected in the increased gelatinisation temperature. Proteins inhibited gelatinisation by forming complex compounds with the starch molecules on the surface of the granules. Due to their hydrophilic nature, proteins inhibit the adsorption by the starch granules, which helps to break the hydrogen bonds between the molecules of starch granule and increasing the gelatinisation temperature (*47*).

## CONCLUSION

The development of a rice analogue using black-eyed beans and the hot extrusion method shows significant improvements in both nutritional and physical properties. The ratio of black-eyed beans to skimmed milk powder has a significant impact on the quality of the rice analogue compared to conventional rice (IR64) and previous studies. Notably, sample formulation 6 showed better results in all quality parameters compared to IR64. These results suggest a nutritionally improved rice substitute. The effect of temperature on the nutrient content of the rice analogues shows changes in protein, fat and moisture mass fraction, indicating a successful fortification process. Increasing the extrusion temperature had an inverse effect on the protein mass fraction but increased the gelatinisation temperature, which ultimately affected the quality of the product. In a sensory evaluation with 30 panellists, the rice analogue was found to be very similar to traditional rice in terms of texture, aroma, taste and colour. The rice analogue that is made from modified sago with the addition of corn and black-eyed beans mimics the characteristics of natural rice (IR64) while offering a higher nutritional content, making it a promising substitute in the face of rice scarcity. These results highlight the potential of black-eyed bean-based rice analogues as a viable alternative for addressing food security challenges. However, further *in vivo* and *in vitro* studies are needed before the product can be widely produced and recommended for consumption. In addition, future studies should perform a comparative analysis of IR64 with the same treatment as the rice analogue. The ingredients used in this rice analogue, including sago, corn and black-eyed beans, are natural resources from Indonesia.

## Appendix

**Table S1:** Research design of raw material composition and temperature process

	*w*/%		
Sample formulation	Black-eyed bean	Skimmed milk	*t*(extrusion)/°C
1	10	0.5	50
2	15	0.5	
3	20	0.5	
4	10	1.0	
5	15	1.0	
6	20	1.0	
7	10	1.5	
8	15	1.5	
9	20	1.5	
10	10	0.5	70
11	15	0.5	
12	20	0.5	
13	10	1.0	
14	15	1.0	
15	20	1.0	
16	10	1.5	
17	15	1.5	
18	20	1.5	
19	10	0.5	90
20	15	0.5	
21	20	0.5	
22	10	1.0	
23	15	1.0	
24	20	1.0	
25	10	1.5	
26	15	1.5	
27	20	1.5	

**Table S2:** Scoring criteria for organoleptic analysis

Parameter	Criterion	Score
Aroma	Very pleasant	4
	Pleasant	3
	Quite pleasant	2
	Unpleasant	1
Texture (compared with conventional rice)	Very similar	4
	Similar	3
	Different	2
	Very different	1
Taste	Very tasteful	4
	Tasteful	3
	Quite tasteful	2
	Untasteful	1
Colour	Very interesting	4
	Interesting	3
	Uninteresting	2
	Very uninteresting	1
